# Genome-Enabled Insights into the Ecophysiology of the Comammox Bacterium “*Candidatus* Nitrospira nitrosa”

**DOI:** 10.1128/mSystems.00059-17

**Published:** 2017-09-12

**Authors:** Pamela Y. Camejo, Jorge Santo Domingo, Katherine D. McMahon, Daniel R. Noguera

**Affiliations:** a Department of Civil and Environmental Engineering, University of Wisconsin—Madison, Madison, Wisconsin, USA; b Environmental Protection Agency, Cincinnati, Ohio, USA; c Department of Bacteriology, University of Wisconsin—Madison, Madison, Wisconsin, USA; ExxonMobil Research and Engineering

**Keywords:** “*Ca.* Nitrospira nitrosa”, comammox, NOB, *Nitrospira*, metagenomics

## Abstract

The recently discovered comammox bacteria have the potential to completely oxidize ammonia to nitrate. These microorganisms are part of the *Nitrospira* genus and are present in a variety of environments, including biological nutrient removal (BNR) systems. However, the physiological traits within and between comammox and nitrite-oxidizing bacterium (NOB)-like *Nitrospira* species have not been analyzed in these ecosystems. In this study, we identified *Nitrospira* strains dominating the nitrifying community of a sequencing batch reactor (SBR) performing BNR under microaerobic conditions. We recovered metagenome-derived draft genomes from two *Nitrospira* strains: (i) *Nitrospira* sp. strain UW-LDO-01, a comammox-like organism classified as “*Candidatus* Nitrospira nitrosa,” and (ii) *Nitrospira* sp. strain UW-LDO-02, a nitrite-oxidizing strain belonging to the *Nitrospira defluvii* species. A comparative genomic analysis of these strains with other *Nitrospira*-like genomes identified genomic differences in “*Ca*. Nitrospira nitrosa” mainly attributed to each strain’s niche adaptation. Traits associated with energy metabolism also differentiate comammox from NOB-like genomes. We also identified several transcriptionally regulated adaptive traits, including stress tolerance, biofilm formation, and microaerobic metabolism, which might explain survival of *Nitrospira* under multiple environmental conditions. Overall, our analysis expanded our understanding of the genetic functional features of “*Ca*. Nitrospira nitrosa” and identified genomic traits that further illuminate the phylogenetic diversity and metabolic plasticity of the *Nitrospira* genus.

**IMPORTANCE** *Nitrospira*-like bacteria are among the most diverse and widespread nitrifiers in natural ecosystems and the dominant nitrite oxidizers in wastewater treatment plants (WWTPs). The recent discovery of comammox-like *Nitrospira* strains, capable of complete oxidation of ammonia to nitrate, raises new questions about specific traits responsible for the functional versatility and adaptation of this genus to a variety of environments. The availability of new *Nitrospira* genome sequences from both nitrite-oxidizing and comammox bacteria offers a way to analyze traits in different *Nitrospira* functional groups. Our comparative genomics analysis provided new insights into the adaptation of *Nitrospira* strains to specific lifestyles and environmental niches.

**Author Video**: An author video summary of this article is available.

## Abstract (precis)

*Nitrospira*-like bacteria are among the most diverse and widespread nitrifiers in natural ecosystems and the dominant nitrite oxidizers in wastewater treatment plants (WWTPs). The recent discovery of comammox-like *Nitrospira* strains, capable of complete oxidation of ammonia to nitrate, raises new questions about specific traits responsible for the functional versatility and adaptation of this genus to a variety of environments. The availability of new *Nitrospira* genome sequences from both nitrite-oxidizing and comammox bacteria offers a way to analyze traits in different *Nitrospira* functional groups. Our comparative genomics analysis provided new insights into the adaptation of *Nitrospira* strains to specific lifestyles and environmental niches.

## INTRODUCTION

Nitrification is a microbiological process that plays an important role in the nitrogen (N) cycle. This process has been conventionally known as a two-step reaction. The first step, oxidation of ammonia to nitrite, is performed by ammonia-oxidizing bacteria (AOB) or archaea (AOA), and the second step, oxidation of nitrite to nitrate, is carried out by nitrite-oxidizing bacteria (NOB). Recently, the discovery of a new player with the potential to completely oxidize ammonia to nitrate, as in the case of complete ammonia-oxidizing (comammox) organisms (1, 2), has dramatically changed our understanding of microbially mediated N transformations in engineered and natural systems.

Comammox bacteria have been classified within the genus *Nitrospira*. Members of this genus were conventionally regarded as NOB and were thought to rely only on nitrite for growth. However, the genomes of the four comammox-like *Nitrospira* bacteria identified to date (“*Candidatus* Nitrospira nitrosa,” “*Candidatus* Nitrospira nitrificans,” “*Candidat*us Nitrospira inopinata,” and *Nitrospira* sp. strain Ga0074138 [1–3]) contain the genes necessary for ammonia and nitrite oxidation, suggesting that *Nitrospira* bacteria are much more metabolically versatile organisms. Furthermore, comammox-like *Nitrospira* bacteria have been identified in a variety of habitats, including groundwater wells, drinking water biofilters, wastewater treatment plants (WWTPs), and other soil and aquatic environments (4). These findings have prompted questions regarding the ecological significance and lifestyle of these organisms in each of these ecosystems.

Nutrient removal in WWTPs relies on nitrifying organisms to remove N from the wastewater. *Nitrospira*-like bacteria appear to be the dominant nitrite oxidizers (5–7) in most WWTPs and laboratory-scale reactors. The abundance of comammox bacteria in WWTPs has been briefly surveyed, and preliminary results show that this functional group is present in these systems (4). However, genetic and functional adaptations of comammox bacteria to this environment have not been addressed.

In this study, the community performing N removal in a biological nutrient removal (BNR) lab-scale reactor was analyzed to explore the genomic basis for comammox ecophysiology. A sequencing batch reactor (SBR) was operated under cyclic anoxic or anaerobic and microaerobic conditions (dissolved oxygen [DO], <0.6 mg/liter) using two different operational stages. During the first stage (nitrite addition during microaerobic phase), two *Nitrospira*-like strains were enriched in the reactor. Draft genome sequences of these two strains were assembled from metagenomic data; one of them was identified as a comammox organism, and the other was identified as an NOB. Here, we used the draft genomes of these strains, as well as genomes from both NOB and comammox-related bacteria, to perform a comparative genome analysis of the genus *Nitrospira*.

## RESULTS AND DISCUSSION

### Nutrient removal in lab-scale reactor.

**FIG 1:**
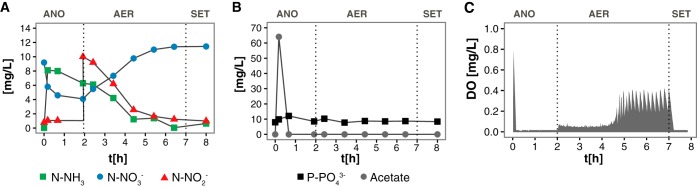
Nutrient profiles of nitrogenous compounds (A), phosphorus and acetate (B), and oxygen concentration (C) in a regular cycle of the lab-scale SBR during stage 1. Dotted lines separate operational conditions within cycle. ANO, anoxic; AER, microaerobic; SET, settling.

Results from a typical cycle of the lab-scale SBR at steady-state operation during stage 1 are shown in Fig. 1. During the first phase, no oxygen was introduced into the system and the presence of nitrate carried over from the previous cycle generated an anoxic environment. Acetate added at the beginning of this phase was completely consumed within an hour (Fig. 1B). Phosphorus (P) release to the mixed liquor during this condition was not observed (Fig. 1B), indicating the absence of polyphosphate-accumulating organisms (PAO) in the reactor. Denitrification was incomplete, with only ∼60% of the nitrate removed in the anoxic phase (Fig. 1A), even though the reactor received acetate in this phase. This suggests that efficient acetate uptake was likely performed by glycogen-accumulating organisms (GAO), without affecting P concentrations (8). In addition, nitrite production during this phase (∼10% of the initial NO_3_^−^ concentration) is an indicator of partial denitrification (Fig. 1A).

Complete nitrification occurred in the subsequent microaerobic stage, where 91% ± 4% of ammonia and 91% ± 8% of the added nitrite were removed (Fig. 1A). With nitrate accumulation accounting for 50% ± 2% of the oxidized nitrogen, the remaining nitrogen was likely denitrified; however, no measurements of NO, N_2_O, or N_2_ were carried out. During the period of active nitrification, the DO remained below 0.05 mg/liter (Fig. 1C), as the oxygen supplied balanced the oxygen uptake rate. The oxygen uptake rate decreased after nitrification ceased, and correspondingly, DO increased. To maintain a low-DO environment, aeration was stopped when DO exceeded the set point (0.2 mg O_2_/liter) and resumed when DO decreased below the set point. This operation effectively maintained DO below 0.4 mg/liter (Fig. 1C).

In summary, operational stage 1 resulted in enhanced nitrification under microaerobic conditions and no P cycling. With a goal of implementing P removal and maintaining low-DO nitrification, after 100 days of reactor operation under stage 1 conditions, the operational parameters were changed by eliminating nitrite addition during microaerobic conditions (stage 2). During this second stage, acetate added at the beginning of the anaerobic phase was used by PAO for P cycling, and nitrite and nitrate produced by ammonia oxidization were used as electron acceptors by PAO during microaerobiosis, achieving simultaneous removal of N and P. Results of this stage were described elsewhere (9).

### *Nitrospira*-like genome binning.

Using a combination of bidimensional coverage and tetranucleotide frequency, two *Nitrospira*-like draft genomes were assembled from a sample collected at the end of stage 1. The two draft genomes (*Nitrospira* sp. strains UW-LDO-01 and UW-LDO-02) had 3.9 and 3.5 Mbp in total with average GC contents of 54.9% and 59.2%, respectively (see Table S1 in the supplemental material). The reconstructed genomes were assessed to be nearly complete (completeness, ≧90%) with low redundancy (≦5%), according to the presence of 43 single-copy reference genes (Table S1).

10.1128/mSystems.00059-17.5 TABLE S1  Metrics of the two *Nitrospira* genomes assembled in this study after each genome refinement step. Download TABLE S1, DOCX file, 0.05 MB. Copyright © 2017 Camejo et al. 2017 Camejo et al. This content is distributed under the terms of the Creative Commons Attribution 4.0 International license.

**FIG 2:**
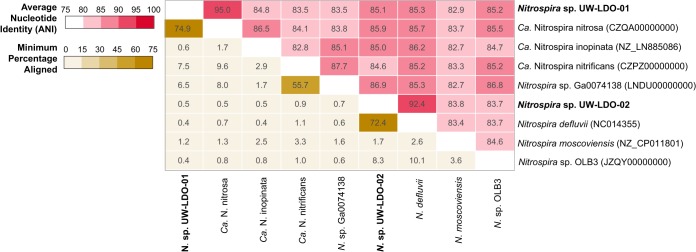
Comparison of the genome-wide average nucleotide identities and alignment percentages of *Nitrospira*-like genomes. The heat map shows the average nucleotide identity (red upper section of matrix) and the percentage of the two genomes that aligned (yellow lower section).

Since the composite genomes did not contain complete 16S rRNA genes, the average nucleotide sequence identity (ANI) between the draft genomes assembled here and formerly published *Nitrospira*-like genomes was used to determine whether UW-LDO-01 and UW-LDO-02 represented distinct species, as this method has been shown to correlate well with previously defined 16S rRNA gene species boundaries (10). The calculated ANI and fraction of alignment for the *Nitrospira* genomes (Fig. 2) showed that UW-LDO-01 is a representative of the “*Ca.* Nitrospira nitrosa” species (ANI, >94%; fraction aligned, 74.9%), while UW-LDO-02 had the closest nucleotide identity to *Nitrospira defluvii* (ANI, 92.4%; fraction aligned, 72.4%). None of the other ANI values were greater than 88%, indicating that the two genomes were different from each other and supporting their classification as “*Ca*. Nitrospira nitrosa” UW-LDO-01 and *Nitrospira defluvii* UW-LDO-02, respectively.

### Phylogenetic analysis.

**FIG 3:**
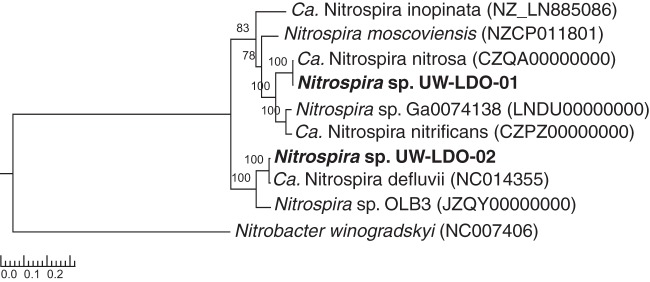
RAxML phylogenetic tree of a concatenated alignment of 37 marker genes (nucleotide sequence) from a data set with the root placed on the branch leading to *Nitrobacter winogradskyi*. The numbers at the nodes of both trees show support values derived from 100 RAxML bootstrap replicates.

A phylogenetic tree constructed from a concatenated protein alignment of 38 universally distributed single-copy marker genes (11) confirms the affiliation of *Nitrospira* sp. UW-LDO-01 and UW-LDO-02 with “*Ca.* Nitrospira nitrosa” and *Nitrospira defluvii*, respectively (Fig. 3). Consistent with this phylogeny, UW-LDO-01 harbored the *amoCAB* operon, responsible for ammonia oxidation, while UW-LDO-02 did not.

The *amoA* and *hao* genes are functional genes involved in redox nitrogen transformations and are also considered phylogenetic markers to study the diversity of ammonia-oxidizing microorganisms (AOM) (12–15). The phylogenetic tree topologies based on these genes (Fig. S1 and S2) further confirm the classification of UW-LDO-01 as related to “*Ca*. Nitrospira nitrosa” (comammox clade A), although two paralogs of the *amoA* gene are present in the genome of “*Ca.* Nitrospira nitrosa,” while only one *amoA* gene was found in UW-LDO-01.

10.1128/mSystems.00059-17.1 FIG S1  Neighbor-joining phylogenetic tree based on full-length nucleotide sequences of homologs to *amoA* in the genome of ammonia-oxidizing prokaryotes, methane-oxidizing bacteria, and comammox. *amoA* sequences identified in other nitrifying prokaryotes through HMM profiling are highlighted in bold and named as the operational day of the sample, contig number in the assembly, and gene position within the contig (start-end). Bootstrap values are shown in the tree branches based on 1,000 bootstrap replicates. The scale bar represents the number of nucleotide substitutions per site. Sequences from *Nitrospira* sp. strain CG24_A-E belong to the genomes assembled in the work of Palomo et al. (37). Download FIG S1, PDF file, 0.3 MB. Copyright © 2017 Camejo et al. 2017 Camejo et al. This content is distributed under the terms of the Creative Commons Attribution 4.0 International license.

10.1128/mSystems.00059-17.2 FIG S2  Neighbor-joining phylogenetic tree based on full-length nucleotide sequences of homologs to *hao* in the genome of ammonia-oxidizing bacteria and comammox. *hao* sequences identified in other nitrifying prokaryotes through HMM profiling are highlighted in bold and named as the operational-day of the sample, contig number in the assembly, and gene position within the contig (start-end). Bootstrap values are shown in the tree branches based on 1,000 bootstrap replicates. The scale bar represents the number of nucleotide substitutions per site. Download FIG S2, PDF file, 0.2 MB. Copyright © 2017 Camejo et al. 2017 Camejo et al. This content is distributed under the terms of the Creative Commons Attribution 4.0 International license.

In addition, the *Nitrospira* sp. UW-LDO-01 and UW-LDO-02 genomes carried the gene for the key enzyme for nitrite oxidation, *nxr*, which can also be used as a phylogenetic biomarker. UW-LDO-01 encoded two paralogs of the periplasmic NXR enzyme while *Nitrospira* UW-LDO-02 carried only one copy. The affiliation of UW-LDO-02 with *N. defluvii* was supported by phylogeny based on the *nxrA* gene sequence (Fig. S3). Likewise, the affiliation of UW-LDO-01 with “*Ca.* Nitrospira nitrosa” was consistent with the phylogenetic analysis of *nxrA*, *amoA*, and *hao* genes.

10.1128/mSystems.00059-17.3 FIG S3  Neighbor-joining phylogenetic tree based on full-length nucleotide sequences of homologs to *nxrA* in the genome of nitrite-oxidizing bacteria and comammox. *nxrA* sequences identified in other nitrifying prokaryotes through HMM profiling are highlighted in bold and named as the operational day of the sample, contig number in the assembly, and gene position within the contig (start-end). Bootstrap values are shown in the tree branches based on 1,000 bootstrap replicates. The scale bar represents the number of nucleotide substitutions per site. Download FIG S3, PDF file, 0.2 MB. Copyright © 2017 Camejo et al. 2017 Camejo et al. This content is distributed under the terms of the Creative Commons Attribution 4.0 International license.

### Nitrifying prokaryotes in lab-scale reactor.

**FIG 4:**
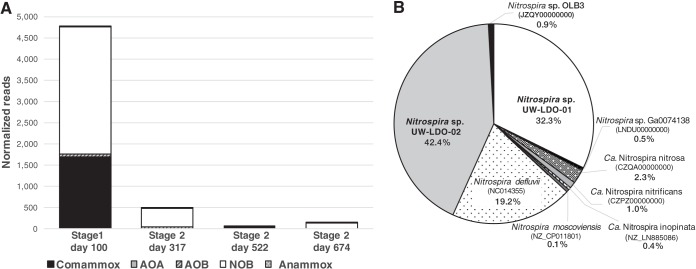
(A) Normalized frequency of metagenomic reads mapping to the genome of comammox organisms, AOA, AOB, NOB, and anammox-related organisms in samples from stages 1 and 2 of the lab-scale SBR. (B) Relative abundance of reads mapping to genomes of *Nitrospira*-related bacteria in stage 1 sample, including the draft genomes retrieved in this study.

The metagenomic analysis of the stage 1 sample, which corresponds to the operational stage under which nitrite and ammonia were both present under microaerobic conditions, did not result in the assembly of any other genome of nitrifying microorganisms. Thus, in order to assess the relative abundance of other known nitrifying prokaryotes present in the reactor, we mapped metagenomic reads to published genomes of comammox and anammox organisms, AOB, AOA, and NOB, including *Nitrospira* sp. UW-LDO-01 and UW-LDO-02 (Fig. 4). After a competitive mapping of short reads from metagenomic samples to each genome (>90% identity), the number of mapping reads was normalized to both metagenome size and reference genome size and used as a proxy of genome abundance.

The metagenomic data show little evidence of AOA and anammox bacteria during the two stages (0.06% and 0.25% of mapping reads, respectively) (Fig. 4A). AOB were detected in the system, albeit representing a small fraction of the community (0.17% and 0.05% of total number of reads during stages 1 and 2, respectively). Notably, *Nitrospira*-like sequences (including comammox- and NOB-like genomes) recruited the greatest number of metagenomic reads (14.0% of total number of reads) in the stage 1 sample (Fig. 4A). Within this genus, *Nitrospira* sp. UW-LDO-01 retrieved 32.3% of the reads competitively mapping to the *Nitrospira*-like genomes (Fig. 4B). The published genome of “*Ca*. Nitrospira nitrosa” retrieved 2.3% of the reads, while less than 4% mapped to other comammox genomes. Therefore, with only a small fraction of reads mapping to other ammonia oxidizers, we propose that *Nitrospira* sp. UW-LDO-01 was the main comammox organism in the reactor and the main contributor to ammonia oxidation during stage 1.

*Nitrospira* sp. UW-LDO-02 appeared to be the most abundant NOB in the reactor during stage 1, retrieving 42.4% of the *Nitrospira*-like reads (Fig. 4B), although a large fraction of reads competitively mapping to *N. defluvii* may indicate the presence of other nitrite-oxidizing strains in the reactor. Therefore, the nitrite oxidation activity in the reactor was carried out by comammox bacteria and NOB.

The metagenomic analysis of stage 2 samples reveals an overall decrease in the relative abundance of nitrifying organisms in the reactor after transitioning to this operational configuration (Fig. 4A). During this stage, metagenomic reads mapping to NOB and comammox genomes (including *Nitrospira* sp. UW-LDO-01 and UW-LDO-02) decreased to less than 1% of the total number of reads. This was in part due to the removal of nitrite addition during stage 2. However, the decrease in comammox bacteria did not correspond to an increase in the number of reads mapping to other known AOM (Fig. 4A), suggesting the presence of still-unrecognized AOM in reactors operated under low-DO conditions, as previously reported (16).

As a confirmation of the results obtained from this analysis and to identify other ammonia and nitrite oxidizers in the reactor, key nitrifying genes (*amoA*, *hao*, and *nxrA*) were searched in assemblies from the four metagenomic samples, using hidden Markov model (HMM) profiling, and the normalized nucleotide coverages of gene-containing contigs were compared for each sample (Fig. S4). Only two contigs containing *amoA* sequences were identified with this analysis, and both of them were assembled from the 100-day sample. The first *amoA* gene corresponded to the one encoded by UW-LDO-01, while the second one clustered with the *amoA* sequence of *Nitrosomonas oligotropha* (Fig. S1). The normalized coverage of these contigs (Fig. S4A) indicates that the two corresponding genomes were present only in samples from stage 1, with the coverage of the UW-LDO-01 *amoA*-containing contig being 6 times higher than that of the contig containing the *amoA* sequence of *N. oligotropha*. Additionally, four *hao* sequences were identified in the metagenome assemblies, one of them belonging to UW-LDO-01 and the other three grouping within the *Nitrosomonas* genus (Fig. S2). Coverage of these contigs showed a similar trend as that of the *amoA*-containing contigs. Specifically, the UW-LDO-01 contig had higher coverage than any other *Nitrosomonas* contig during stage 1 but disappeared during stage 2 with no comparable increment in the coverage of *Nitrosomonas*. In the case of *nxrA*, five contigs containing sequences of this gene were identified using HMM profiling. Two of these sequences belong to UW-LDO-01, and one belongs to UW-LDO-02. One of the other *nxrA* genes was phylogenetically associated with *Nitrospira defluvii* and had a contig coverage similar to that of the *nxrA*-containing contig in UW-LDO-02. Thus, it likely corresponds to a second *nxrA* copy missed in the assembly of this genome. The last *nxrA* gene sequence identified here did not cluster with any specific *Nitrospira* species and displayed the lowest contig coverage in all samples analyzed. As was observed for the *amoA* and *hao* genes, the *nxrA* gene of UW-LDO-01 disappeared after stage 1 and the coverage of the other *nxrA*-containing genes was drastically reduced after this stage (Fig. S4C). This analysis confirms the results presented in Fig. 4A, showing the disappearance of comammox bacteria in the reactor and a decrease in the population of NOB after stage 1, with no meaningful increment in AOB.

10.1128/mSystems.00059-17.4 FIG S4  Normalized metagenomic read coverage of contigs containing *amoA* (A), *hao* (B), and *nxrA* (C) genes, identified using HMM profiling, in samples from stages 1 and 2 of the lab-scale SBR. Sequences not belonging to UW-LDO-01 and UW-LDO-02 are labeled according to the sample day, contig number in the assembly, and position within contig (start-end). Download FIG S4, PDF file, 0.2 MB. Copyright © 2017 Camejo et al. 2017 Camejo et al. This content is distributed under the terms of the Creative Commons Attribution 4.0 International license.

Despite some studies pointing to low DO as a strategy to reduce NOB population growth, based on the higher oxygen affinity of AOB than of NOB (17–20), here we show the prevalence of *Nitrospira* species in a system operated under low-DO conditions, as also reported in other studies (1, 2, 16, 21–23). Overall, these studies demonstrate that the single limitation of oxygen supply does not always lead to NOB suppression. Nevertheless, the population of *Nitrospira* decreased when the population of denitrifying PAO increased in the reactor (9), possibly indicating outcompetition of these microorganisms under microaerobic conditions and higher oxygen affinity.

### **Differential gene content among “*****Ca.*** Nitrospira nitrosa” **genomes.**

Since *Nitrospira* sp. UW-LDO-01 is the second comammox genome representative of “*Ca.* Nitrospira nitrosa” and the first comammox genome recovered from a nutrient removal bioreactor, a comparative analysis of its genetic content was carried out. First, a comparison of gene content between “*Ca.* Nitrospira nitrosa” (CZQA00000000) and *Nitrospira* sp. UW-LDO-01 was conducted by blastp comparison of the translated coding DNA sequence (CDS) set, clustering of ortholog proteins, and annotation of representatives of each ortholog cluster (OC) and genome-unique CDS.

**FIG 5:**
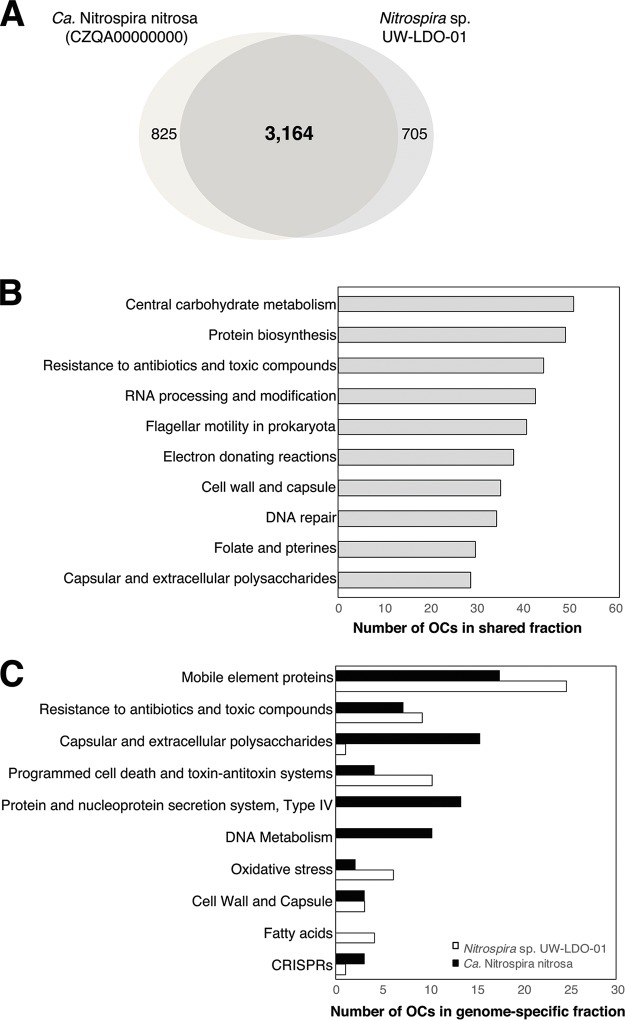
Genomic differences between *Nitrospira* sp. UW-LDO-01 and “*Ca*. Nitrospira nitrosa.” (A) Venn diagram of ortholog clusters shared between the two draft genomes; (B) distribution of SEED functional categories in the fraction of orthologs shared by the two genomes; (C) distribution of SEED functional categories in ortholog clusters found in only one of the genomes (genome-specific fraction).

Overall, sequencing and annotation of the UW-LDO-01 genome revealed a genomic inventory highly similar to the genome of “*Ca.* Nitrospira nitrosa” (2). The two genomes shared 67% of the OCs (3,164 OCs), with UW-LDO-01 and “*Ca.* Nitrospira nitrosa” having 705 and 825 unique OCs, respectively (Fig. 5A).

OCs belonging to the shared and genome-specific fractions of UW-LDO-01 and “*Ca.* Nitrospira nitrosa” were classified according to their predicted functional role, using the SEED level 2 categories (Fig. 5B and C). The majority of OCs were classified as hypothetical proteins (35% of shared OCs and 64% and 62% of the genome-specific fractions in UW-LDO-01 and “*Ca.* Nitrospira nitrosa,” respectively), indicating a large set of metabolic features not yet elucidated. Twenty-eight percent of OCs were classified only at the role hierarchy level but were not assigned to any level 2 category.

OCs within the shared fraction were mostly represented by proteins classified as “central carbon metabolism,” “protein biosynthesis,” and “electron donating reactions,” indicating general conservation of energy metabolism. The “resistance to antibiotics and toxic compounds” category was also highly represented in both the shared and genome-specific fractions. The presence of these genes in the genome of “*Ca.* Nitrospira nitrosa” and UW-LDO-01 can increase the fitness of this species in some environments, facilitating colonization of new niches.

In both genomes, the functional group “mobile element proteins” was overrepresented within the genome-specific fraction. This functional category includes transposases, integrases, and other mobile genetic elements. Likewise, both genomes encoded multiple copies of toxin-antitoxin (TA) systems (*higA*/*higB* and *phd*/*doc* cassettes). Genes encoding these proteins are recognized to be part of the mobile genome and involved in the maintenance of these integrated mobile genetic elements (24). Extensive representation of these traits in the genome-specific fraction indicates that horizontal gene transfer has likely played a significant role in the diversification of “*Ca*. Nitrospira nitrosa” strains.

Type IV secretion system proteins, specifically proteins belonging to the VirB/D operons (25, 26), were enriched in the genome-specific fraction of “*Ca.* Nitrospira nitrosa” but absent in UW-LDO-01. This complex is responsible for transporting macromolecules out of Gram-negative bacteria, including conjugal transfer of plasmids between microorganisms and secretion of virulence factors into the extracellular environment. In “*Ca.* Nitrospira nitrosa,” these sets of proteins are adjacent to a genomic region containing plasmid-related genes (TraP, TraO, and TrbM) and a toxin-antitoxin system (*higA/higB*), as well as a genomic island (identified with IslandViewer 4 [27]). These results suggest the potential genetic mobility of this system and would explain why it is not encoded in the genome of UW-LDO-01.

The greatest difference among the genome-specific fractions of UW-LDO-01 and “*Ca.* Nitrospira nitrosa” was the proportion of OCs represented by the “capsular and extracellular polysaccharides” (Fig. 5C). Glycosyltransferases (28–31) and other enzymes involved in formation of polysaccharide (main component of the biofilm matrix) were enriched within this SEED category in the genome-specific fraction of “*Ca*. Nitrospira nitrosa.” Differences in biofilm formation capabilities between these strains may relate to specific niche adaptation: “*Ca.* Nitrospira nitrosa” was enriched in a biofilm, whereas UW-LDO-01 was found in a planktonic habitat in wastewater. Analogous findings have been observed in other genera, where differences among biofilm formation capabilities within the same genus were linked to the genome content of different strains (32–34). Similarly to the results presented here, these genetic differences included the presence of type IV secretion systems and enzymes involved in protein glycosylation.

The comparative genomic analysis also indicated a higher proportion of gene clusters associated with “fatty acids” in *Nitrospira* sp. UW-LDO-01 (Fig. 5C). Genes related to β-oxidation of long-chain fatty acids to acetyl coenzyme A (acetyl-CoA) were present in the genome of UW-LDO-01 but absent in “*Ca.* Nitrospira nitrosa.” These genes include a long-chain fatty-acid–CoA ligase, acyl-CoA dehydrogenase, enoyl-CoA hydratase, 3-hydroxyacyl–CoA dehydrogenase, and acetyl-CoA acetyltransferase. The presence of these lipid-related metabolic genes in other *Nitrospira* strains was confirmed, although the complete pathway is lacking in *Nitrospira defluvii*, “*Ca.* Nitrospira nitrificans,” and *Nitrospira* sp. Ga0074138. This feature may represent a competitive advantage of some *Nitrospira* strains in habitats rich in long-chain fatty acids, such as WWTPs (35).

Finally, since the two genomes analyzed here correspond to draft sequences, it is possible that individual genes may be missing in the assemblies.

### Metabolic features in *Nitrospira* genomes.

To explore the diverse metabolic capabilities and provide insights into the common and unique metabolic features encoded in the genomes of NOB- and comammox-like strains, we compared the gene inventories of 9 complete and draft genomes classified as *Nitrospira*. The analysis was focused on traits associated with energy production, which are summarized in Table S3.

In agreement with previous analyses, only comammox-like genomes harbored ammonia monooxygenase (*amoCAB*) and hydroxylamine dehydrogenase (*haoAB-cycAB*) gene clusters, responsible for oxidation of ammonia to nitrite (Table S3), reflecting the capability of this novel *Nitrospira* sublineage to perform full nitrification from ammonia to nitrate.

Analysis of nitrite-reducing genes revealed that all *Nitrospira* strains encoded a copper-containing dissimilatory nitrite reductase (*nirK*), which catalyzes the reduction of nitrite to nitric oxide, a key step in the denitrification process. Despite the widespread presence of this enzyme across the *Nitrospira* genus, former studies have documented no activity of this protein in NOB-like (36) or comammox-like (1) strains, where N loss caused by formation of gaseous compounds was not observed. Since it has been predicted that the NXR complex of *Nitrospira* can reduce nitrate to nitrite (36), these microorganisms appear genetically capable of converting nitrate (the product of nitrification) to nitric oxide. Additional experiments are still needed to obtain more insights into this *Nitrospira* trait. Other denitrification genes, such as those for nitrate reductase (*nar*), nitric oxide reductase (*nor*), or nitrous oxide reductase (*nos*), were not found in the *Nitrospira* strains analyzed here.

All comammox-like genomes, including UW-LDO-01, encoded the machinery to hydrolyze urea, the *ureABCDFG* urease operon and the *urtABCDE* urea transport system, suggesting that this *Nitrospira* subdivision possesses a high-affinity uptake system for urea and, thus, is adapted to habitats where urea is present at low levels. Similar findings have been reported by Palomo et al. (37), during analysis of other comammox-classified metagenome assembled draft genomes. A gene cluster involved in urea metabolism was also found in *Nitrospira moscoviensis* (Table S3), although the urea-binding protein genes *urtBCDE* were lacking in the genome. Ureolytic activity of *N. moscoviensis*, “*Ca.* Nitrospira nitrosa,” and “*Ca*. Nitrospira nitrificans” was formerly tested by incubation of these strains with urea-containing media, where urea hydrolysis to ammonium was observed in both cases (2, 36). Former studies have also shown the presence of genes for urea utilization in *Nitrospira lenta* (38), a novel *Nitrospira* species enriched under low temperatures, suggesting that the ureolytic activity might be associated with lineage II.

A contrasting difference among NOB-like and comammox-like genomes was the capability to convert cyanate into ammonia. Only NOB-like genomes encoded a cyanase hydratase enzyme, and former studies have experimentally confirmed cyanate degradation in *N. moscoviensis* (39). Cyanate is produced intracellularly from urea and carbamoyl phosphate decomposition (40, 41) and in the environment from the chemical/physicochemical decomposition of urea or cyanide (42, 43). The presence of a cyanase enzyme benefits nitrite oxidizers because it allows them to detoxify cyanate, and the formed ammonium is then available for assimilation and might also serve as a source of energy for ammonia oxidizers in a process described as “reciprocal feeding” (36, 39). Further experiments analyzing the effect of cyanate in the growth on comammox-like bacteria are needed to understand how cyanate degradation would give them a biological advantage, besides generation of ammonia.

The analysis also revealed the presence of the gene inventory for the uptake and oxidation of formate, an exclusive feature of NOB (37). Growth on formate as an electron donor has been confirmed in *N. moscoviensis* (under both microoxic and anoxic incubations) (36), *Nitrospira japonica* (44), and uncultured *Nitrospira* in activated sludge (45). Despite formate oxidation potentially being an advantageous feature for organisms thriving in hypoxic or anoxic habitats, since it is a common end product of bacterial fermentation, this feature has not been found in the genome of comammox-like bacteria.

The genome of *N. moscoviensis* encodes a group 2a [Ni-Fe] hydrogenase (*hupS* and *hupL*) and accessory proteins involved in the maturation and transcriptional regulation of hydrogenases (*hypFCDEAB* and *hoxA*). Furthermore, experiments showed that *N. moscoviensis* was capable of growing by aerobic respiration of H_2_ (46). Although the comammox-like genomes lack the subunits of the [Ni-Fe] hydrogenase (Hup), the five genomes analyzed here, as well as the comammox clade A draft genomes in the work of Palomo et al. (37), contained a group 3 [Ni-Fe] sulfur-reducing hydrogenase gene set (*hydBGDA* and *hybD*) positioned at the same locus where Hup is located in *N. moscoviensis*. This hydrogenase complex is a heterotetramer with both hydrogenase activity and sulfur reductase activity, which might play a role in hydrogen cycling during fermentative growth (47). Its beta and gamma subunits, which form the sulfur-reducing component, catalyze the cytoplasmic production of hydrogen sulfide in the presence of elemental sulfur. The presence of this complex in the genomes indicates the potential of these microorganisms for oxidizing H_2_ using sulfur as an electron acceptor, a trait that has not been analyzed in comammox bacteria before but that could give this subgroup an advantage when growing under anaerobic conditions.

Furthermore, the presence of a *hyf*-like operon (*hyfBCEFGI*), which encodes a putative group 4 hydrogenase complex, was detected in every NOB-like genome, as well as “*Ca.* Nitrospira nitrosa,” *Nitrospira* sp. Ga0074138, and UW-LDO-01. This operon was also contained in another recently assembled comammox draft genome (37). In *Escherichia coli*, this hydrogenase complex forms part of a second formate hydrogen lyase pathway (oxidation of formate to CO_2_ and reduction of 2H^+^ to H_2_ under fermentative conditions) (48). This is likely the case for the hydrogenase-4 present in the genome of NOB-like strains, which cooccurs with genes encoding formate dehydrogenase. In comammox bacteria, however, the role of this distinct hydrogenase is not as clear. In “*Ca.* Nitrospira nitrosa,” this complex is found immediately adjacent to a carbon monoxide dehydrogenase (CODH), an enzyme that catalyzes the interconversion of CO and CO_2_ (49), a genomic feature that would allow this strain to obtain energy from carbon monoxide (50). Conversely, the genomes of *Nitrospira* sp. Ga0074138 and UW-LDO-01 lack the CODH at this position, which in the case of UW-LDO-01 was confirmed by alignment of the metagenomic reads to this gene. No other neighboring gene of the hydrogenase-4 complex could be associated with this enzyme in these two strains; therefore, the biological role of these genes is still unclear.

Altogether, these results reveal specific traits characterizing the NOB and comammox functional groups: while comammox-like *Nitrospira* has the genomic potential of ammonia and nitrite oxidation and potentially sulfur reduction, NOB-like strains are distinguished by their cyanate degradation and formate oxidation capabilities, and both urea hydrolysis and H_2_ respiration are common traits shared by multiple *Nitrospira* strains.

### The role of transcriptional regulation in *Nitrospira.*

Transcriptional regulation of gene expression is the most commonly used strategy to control many of the biological processes in an organism, including progression through the cell cycle, metabolic and physiological balance, and responses to environmental stress. This regulation is generally orchestrated by several transcriptional factors (TFs) that directly coordinate the activity of genes by binding to their promoters. Each *Nitrospira*-like genome codes for at least 100 transcriptional regulators, which account for ∼3% of the estimated total number of genes, in agreement with TFs in other microorganisms (51–53). A comparative genomic analysis of full and draft *Nitrospira* genomes was used to investigate the repertoire of TFs potentially involved in the survival of these microorganisms under diverse environmental conditions (Table 1).

**TABLE 1:** Inventory of transcriptional regulators with implications for adaptive metabolism, from complete and draft genomes of *Nitrospira*

Transcriptional regulator	Gene	Presence or absence of gene in species or strain^a^									Function
		Comammox organisms					NOB				
		*Nitrospira* sp. UW-LDO-01	“*Ca.* Nitrospira nitrosa”	“*Ca.* Nitrospira nitrificans”	“*Ca.* Nitrospira inopinata”	*Nitrospira* sp. Ga0074138	*Nitrospira* sp. UW-LDO-02	*Nitrospiramoscoviensis*	*Nitrospiradefluvii*	*Nitrospira* sp. OL23	
Formate hydrogen lyase transcriptional activator	*fhlA*	+	+	+	+	+	+	+	+	+	FhlA binds to formate hydrogen lyase structural genes (formate dehydrogenase and group 4 hydrogenase complex) to activate transcription of their promoters (54).
Transcriptional activator protein NhaR	*nhaR*	+	+	+	+	+	+	+	−	−	NhaR regulates *nhaA*, a pH-dependent sodium-proton antiporter that responds to alkaline and saline conditions (82). It is also responsible for *osmC* induction (55), required for resistance to organic peroxides and osmotic conditions and for long-term survival in stationary phase (83, 84). NhaR also stimulates transcription of *pga*, a set of genes responsible for poly-β-1,6-*N*-acetyl-d- glucosamine (PGA) synthesis (56). PGA is involved in cell-cell adhesion and attachment, which stabilize biofilm formation (85).
Hydrogen peroxide-inducible gene activator	*oxyR*	+	+	+	+	+	+	−	+	+	OxyR is required for the induction of a hydrogen peroxide-inducible regulon in response to elevated levels of hydrogen peroxide (57).
Chemotaxis regulator CheZ	*cheZ*	+	+	+	+	+	−	+	+	+	CheZ is a component of the chemotaxis signal-transduction pathway (86). It controls the phosphorylation of CheY, a protein involved in the cell excitation response. Absence of CheZ results in nonchemotactic cells or long stimulus response latencies, demonstrating its critical importance during response to stimuli (87).
Fumarate and nitrate reductase regulatory protein	*fnr*	+	+	+	−	−	+	+	+	+	Fnr is an oxygen-responsive regulator required for the expression of a number of genes involved in anaerobic metabolism (61, 88, 89), including fumarate reductase, nitrate and nitrite reductase, and cytochrome oxidase genes (90).

a Plus and minus signs represent the presence and absence of each gene, respectively.

Among the TFs analyzed, the formate hydrogen lyase transcriptional activator (FhlA) (48, 54) was the only one shared across all the *Nitrospira* genomes, although only NOB-like genomes contain genes of its known regulon, the formate hydrogenase complex. The presence of this transcriptional activator in comammox microorganisms, which appear to be genetically incapable of formate oxidation (Table 1), might represent an ancestral trait shared by *Nitrospira* and lost during diversification. This theory would also support the presence of the group 4 hydrogenase (associated with the formate-hydrogen lyase complex in *E. coli*) in both NOB- and comammox-like groups.

A common feature among some NOB and comammox bacteria is the presence of the transcriptional regulators NhaR (55, 56) and OxyR (57) (Table 1). The first one is associated with the stress response to alkaline, acidic, saline, and osmotic conditions. OxyR regulates hydrogen peroxide-inducible genes, such as alkyl hydroperoxide reductase (*ahpCF*) and glutaredoxin (*grxA*), carried in all the *Nitrospira* genomes. The presence of these genes would give *Nitrospira* an improved fitness advantage over other nitrifying bacteria. For instance, NhaR is lacking in *Nitrosomonas* and *Nitrobacter* and OxyR is not present in *Nitrosomonas* and *Nitrosospira* (based on genome searching). Furthermore, the role of NhaR during regulation of *pga* expression (56) allows the biofilm formation process to be considered a flexible and dynamic developmental process driven by external conditions, representing another means by which NhaR could promote survival of *Nitrospira*. Likewise, the presence of the chemotaxis regulator CheZ in *Nitrospira* suggests chemotaxis as another important mechanism by which these microorganisms efficiently and rapidly respond to changes in the chemical composition of their environment.

To date, the role of the Fnr-type regulatory protein in *Nitrospira* has not been determined. In other microorganisms, Fnr is part of the signaling involved in the adaptation to microoxic environments (58–62), where it acts as an oxygen sensor and regulator of genes involved in anaerobic and microaerobic metabolism. In *Nitrospira*, we predict that this TF would regulate similar genes, such as the *frd* operon (fumarate reductase), *sdh* operon (succinate dehydrogenase), *ndh* (NADH dehydrogenase), and *ccb3* complex (cytochrome *c* oxidase). At least one copy of Fnr in the genomes of UW-LDO-01, *N. moscoviensis*, *N. defluvii*, and *Nitrospira* sp. strain OLB3 was located upstream of a copper-containing nitrite reductase gene (*nirK*), suggesting a possible mechanism that controls expression of this denitrification enzyme. The presence of multiple paralog copies of Fnr in several *Nitrospira* genomes may indicate a rigorous regulation of metabolism when these microorganisms are exposed to low levels of oxygen, an important factor affecting *Nitrospira* community compositions in nitrifying systems (23).

Overall, this study sheds light on differences in the physiological roles of NOB and comammox-like *Nitrospira*. Specifically, the comparative genomic results show traits associated with energy metabolism as characteristic of each of these functional groups. Furthermore, the analysis of TFs in *Nitrospira* reveals the alternative use of organic compounds, response to environmental stress, chemotaxis, and anaerobic metabolism as some of the key mechanisms for the adaptive metabolism of the genus to multiple and adverse conditions. Further studies in the field should include experiments that combine omics analysis (transcriptomics, metabolomics, and proteomics) with chemical data to confirm the ecological role and functionality of each of these functional groups and their interactions with other microorganisms.

## MATERIALS AND METHODS

### Operation of lab-scale sequencing batch reactor.

A laboratory-scale SBR was originally inoculated with activated sludge obtained from the Nine Springs WWTP in Madison, WI, which uses a modified University of Cape Town (UCT) process designed to achieve biological P removal (63) and operates with high aeration rates (64). Synthetic wastewater containing acetate as the sole carbon source was used for the feed, as described elsewhere (9). The hydraulic retention time (HRT) and solid retention time (SRT) were 24 h and 80 days, respectively. The pH in the system was controlled to be between 7.0 and 7.5.

The 2-liter reactor was operated under alternating anoxic or anaerobic and low-oxygen cycles. During stage 1 of operation, the cycles consisted of 2 h of anoxic conditions, 5 h of microaerobic conditions, 50 min of settling, and 10 min of decanting. At the beginning of the microaerobic phase, sodium nitrite was added to reach an in-reactor concentration of 10 mg N-NO_2_^−^/liter to potentially stimulate the use of nitrite as an electron acceptor by denitrifying PAO. In addition, an on/off control system was used to limit the amount of oxygen pumped to the reactor (0.02 liters/min) and maintain low dissolved oxygen (DO) concentrations in the mixed liquor, as described elsewhere (9). After 100 days of operation, the nitrite supplement was eliminated and the reactor cycle was changed to: 1.5 h of anaerobic conditions, 5.5 h of microaerobic conditions, 50 min of settling, and 10 min of decanting (stage 2).

### Sample collection and analytical tests.

To monitor reactor performance, mixed liquor and effluent samples were collected, filtered through a membrane filter (0.45 μm; Whatman, Maidstone, United Kingdom), and analyzed for acetate, PO_4_^3−^-P, NH_4_^+^-N, NO_3_^−^-N, and NO_2_^−^-N. The concentrations of PO_4_^3−^-P were determined according to standard methods (65). Total ammonia (NH_3_ + NH_4_^+^) concentrations were analyzed using the salicylate method (method 10031; Hach Company, Loveland, CO). Acetate, nitrite, and nitrate were measured using high-pressure liquid chromatography as previously described (9).

Seven milliliters of biomass samples from the reactors was collected weekly and stored in 15% glycerol at −80°C until DNA extraction was performed. DNA was extracted using the UltraClean soil DNA isolation kit (Mo Bio Laboratories, Carlsbad, CA). Extracted DNA was quantified using a NanoDrop spectrophotometer (Thermo Fisher Scientific, Waltham, MA) and stored at −80°C.

### Metagenome sequencing, assembly, and binning.

Samples from day 100 (stage 1) and days 317, 522, and 674 (stage 2) were selected for metagenomic analysis. Illumina TruSeq DNA PCR free libraries were prepared for DNA extracts according to the manufacturer’s protocol and paired-end sequenced on either the Illumina HiSeq 2000 platform (v4 chemistry; 2 by 150 bp; 522-day sample) or the Illumina MiSeq platform (v3 chemistry; 2 by 250 bp; other samples). This sequencing method generated 1.7, 2.1, 16.2, and 2.4 gigabases (Gb) of data for 100-, 317-, 522-, and 674-day samples, respectively. Unmerged reads were quality trimmed and filtered with Sickle (https://github.com/ucdavis-bioinformatics/sickle.git) using a minimum Phred score of 20 and a minimum length of 50 bp. The metagenomic reads from each sample were assembled using IDBA-UD (66). Individual genome bins were extracted from the metagenome assembly from the 100-day sample (stage 1) with the R package “mmgenome” (67) using the differential coverage principle (68). The bins were initially extracted by plotting the genome coverage of contigs in metagenomes from days 100 and 317. During the bin extraction, GC content and taxonomy of contigs were also taken into consideration.

After binning, SSPACE was used to filter small scaffolds (length, <1,000 bp), extend scaffolds, and fill gaps (69). Genome completeness and contamination were estimated using CheckM 0.7.1 (70). Table S1 in the supplemental material displays quality metrics of the draft genomes after each of the steps previously described. Two putative *Nitrospira*-like bins were identified and annotated using MetaPathways v2.0 (71) and rapid annotation using subsystem technology (RAST) (72). To further reduce contamination in these assembled bins, scaffolds containing open reading frames (ORFs) with 0% protein identity or less than 85% nucleotide identity to other *Nitrospira* genomes were removed from the bins.

### ANI.

Pairwise average nucleotide identity (ANI) values of *Nitrospira-*like genomes were obtained using the ANIm method (73) and implemented in the Python script “calculate_ani.py” available at https://github.com/ctSkennerton/scriptShed/blob/master/calculate_ani.py.

### Phylogenetic analyses.

The phylogeny of the draft genomes was assessed by constructing a phylogenetic tree using a concatenated alignment of marker genes. First, PhyloSift v1.0.1 (74) was used to extract a set of 38 marker genes from each genome. Then, the extracted marker protein sequences were concatenated into a continuous alignment to construct a maximum-likelihood (ML) tree, using RAxML v7.2.8 (75). RAxML generated 100 rapid bootstrap replicates followed by a search for the best-scoring ML tree.

For phylogenetic analyses of ammonia monooxygenase subunit A (*amoA*), hydroxylamine reductase (*hao*), and nitrite oxidoreductase subunit A (*nxrA*) genes, full nucleotide data sets were downloaded from the NCBI GenBank database (76). A total of 85 *amoA*, 33 *hao*, and 42 *nxrA* sequences were aligned with the genes encoded in the draft genomes. Alignment was performed using the “AlignSeqs” command in the DECIPHER “R” package (77). Phylogenetic trees were calculated using the neighbor-joining criterion with 1,000 bootstrap tests for every node, using the MEGA6 software package (78). Trees were visualized with the assistance of TreeGraph (79).

### Population structure by metagenomic analysis.

To estimate the abundance of currently known ammonia oxidizers, comammox, and nitrite oxidizers in the reactor over time, paired-end DNA reads from the metagenomic data sets (days 100, 317, 522, and 674) were competitively mapped to the published genome sequences of 14 AOB (*Nitrosomonas*, *Nitrosospira*, and *Nitrosococcus* genera), 6 AOA (*Nitrososphaera*, *Nitrosoarchaeum*, and *Nitrosopumilus*), 5 NOB (*Nitrospira* and *Nitrobacter* lineage), 5 anaerobic ammonia-oxidizing (anammox) bacteria (“*Candidatus* Brocadia fulgida,” “*Candidatus* Brocadia caroliniensis,” “*Candidatus* Kuenenia stuttgartiensis,” “*Candidatus* Brocadia sinica,” and “*Candidatus* Jettenia caeni”), 4 comammox bacteria (“*Ca.* Nitrospira nitrosa,” “*Ca.* Nitrospira nitrificans,” “*Ca.* Nitrospira inopinata,” and *Nitrospira* sp. Ga0074138), and the two *Nitrospira*-like draft genomes retrieved from the reactor, using the software package BBMap version 35.85 (https://sourceforge.net/projects/bbmap). A list of the genomes included in this analysis and the number of reads mapping to each sequence are found in Table S2. For each organism, the number of unambiguous reads (best hit) mapping to the genomic sequence with a minimum alignment identity of 90% was quantified and normalized as (total number of mapped reads) × (paired-end read average length)/(number of metagenomics reads) (genome size) (Table S2).

10.1128/mSystems.00059-17.6 TABLE S2  Genomes included in the metagenomic mapping analysis and the number of reads mapping to each of them. The number of mapping reads was also normalized by the number of reads in each metagenome, paired-end read average length, and genome size. Download TABLE S2, DOCX file, 0.1 MB. Copyright © 2017 Camejo et al. 2017 Camejo et al. This content is distributed under the terms of the Creative Commons Attribution 4.0 International license.

10.1128/mSystems.00059-17.7 TABLE S3  Inventory of genes involved in energy-driving processes from complete and draft genomes of *Nitrospira*. Gray and white rectangles represent the presence and absence of each gene, respectively. Download TABLE S3, DOCX file, 0.1 MB. Copyright © 2017 Camejo et al. 2017 Camejo et al. This content is distributed under the terms of the Creative Commons Attribution 4.0 International license.

### HMM gene profiling.

Alignments of sequences from *amoA*, *hao*, and *nxrA* carried in different AOB, NOB, and comammox species were used to create a profile hidden Markov model (HMM) for each gene using “hmmbuild” in the HMMER package (80). These models were used to search homolog genes in contigs from each assembly, by using the “hmmbuild” command (E value, >0.01). Genes identified in this analysis were filtered by length (>50% average gene length) and included in phylogenetic trees constructed for each gene (see “Phylogenetic analyses”). Gene sequences that did not phylogenetically cluster with nitrifying prokaryotes were also removed from the analysis. Then, paired-end DNA reads from each metagenomic data set were competitively mapped to each assembly, using the software package BBMap version 35.85 (https://sourceforge.net/projects/bbmap), and the coverage (average fold) of each contig containing nitrifying genes identified through HMM profiling was normalized by metagenome length.

### Orthologous gene clusters.

To assess the degree of homology in the proteomes of the two *Nitrospira*-like genomes, orthologous gene clusters (OCs) were determined using OrthoMCL (81). OrthoMCL was run with a BLAST E value cutoff of 1e−5 and an inflation parameter of 1.5. Protein products of each ortholog set were classified according to the functional assignment based on SEED subsystem hierarchical levels.

### Accession number(s).

Raw reads and draft genome sequences have been submitted to NCBI and are accessible under the BioProject identifier PRJNA322674.

10.1128/mSystems.00059-17.8 DATA SET S1  Fasta file with contigs from the assembled metagenomic data containing nitrifying genes (*amoA*, *hao*, and *nxrA*). Download DATA SET S1, TXT file, 0.01 MB. Copyright © 2017 Camejo et al. 2017 Camejo et al. This content is distributed under the terms of the Creative Commons Attribution 4.0 International license.
