# Supplementary material for: Genome-Enabled Insights into the Ecophysiology of the Comammox Bacterium “Candidatus Nitrospira nitrosa”
Source: mSystems. 2017 Sep 12;2(5):e00059-17. doi: 10.1128/mSystems.00059-17 (PMC5596200; doi:10.1128/mSystems.00059-17)
Supplement: TABLE S1 [file sys005172133st1.docx]

**Table S1.** Metrics of the two *Nitrospira* genomes assembled in this study after each genome refinement step.

| Bin Id | Assembly Step | GC (%) | Genome size (Mbp) | Gene count | # Scaffolds | N50 | Completeness (%) | Contamination  (%) | Strain heterogeneity (%) |
| --- | --- | --- | --- | --- | --- | --- | --- | --- | --- |
| UW-LDO-01 | MMgenome | 55.1 | 4.13 | 4479 | 523 | 17,227 | 95.9 | 3.79 | 0 |
|  | Contigs length >1,000 | 55.0 | 4.08 | 4330 | 422 | 17,431 | 95.9 | 3.79 | 0 |
|  | SSpace Scaffolding | 55.0 | 4.09 | 4268 | 287 | 27,733 | 95.9 | 3.74 | 0 |
|  | SSpace Gap Filling | 55.0 | 4.08 | 4247 | 287 | 27,614 | 95.9 | 3.74 | 0 |
|  | Removal of scaffolds with non-*Nitrospira* ORFs | 54.9 | 3.91 | 4031 | 230 | 29,398 | 95.9 | 3.64 | 0 |
| UW-LDO-02 | MMgenome | 59.3 | 4.17 | 5360 | 1,878 | 4,227 | 97.5 | 15.4 | 0 |
|  | Contigs length >1,000 | 59.2 | 3.69 | 4262 | 1,003 | 4,891 | 94.3 | 6.10 | 0 |
|  | SSpace Scaffolding | 59.2 | 3.72 | 4082 | 584 | 10,904 | 94.3 | 4.66 | 0 |
|  | SSpace Gap Filling | 59.2 | 3.70 | 3993 | 584 | 10,895 | 94.4 | 4.66 | 0 |
|  | Removal of scaffolds with non-*Nitrospira* ORFs | 59.2 | 3.54 | 3809 | 524 | 11,000 | 94.4 | 4.66 | 0 |

Completeness, contamination and strain heterogeneity were assessed with CHECKM 0.7.1, according to the presence of 43 single-copy

reference gene
