# Supplementary figures and images for: Genome-Enabled Insights into the Ecophysiology of the Comammox Bacterium “Candidatus Nitrospira nitrosa”
Source: mSystems. 2017 Sep 12;2(5):e00059-17. doi: 10.1128/mSystems.00059-17 (PMC5596200; doi:10.1128/mSystems.00059-17)

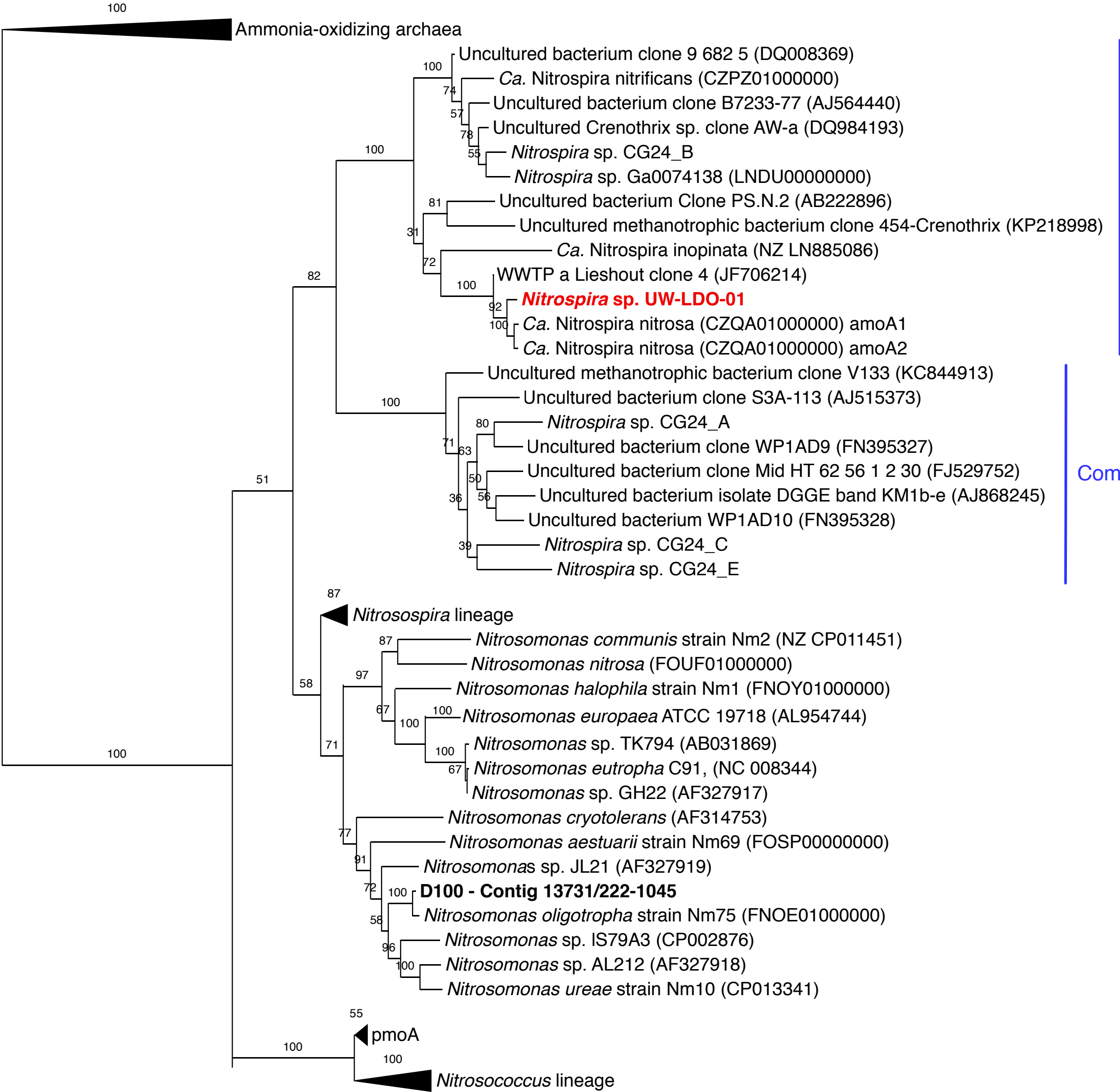

Comammox Clade A

Comammox Clade B

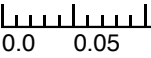

Supplement: FIG S1 [file sys005172133sf4.pdf]

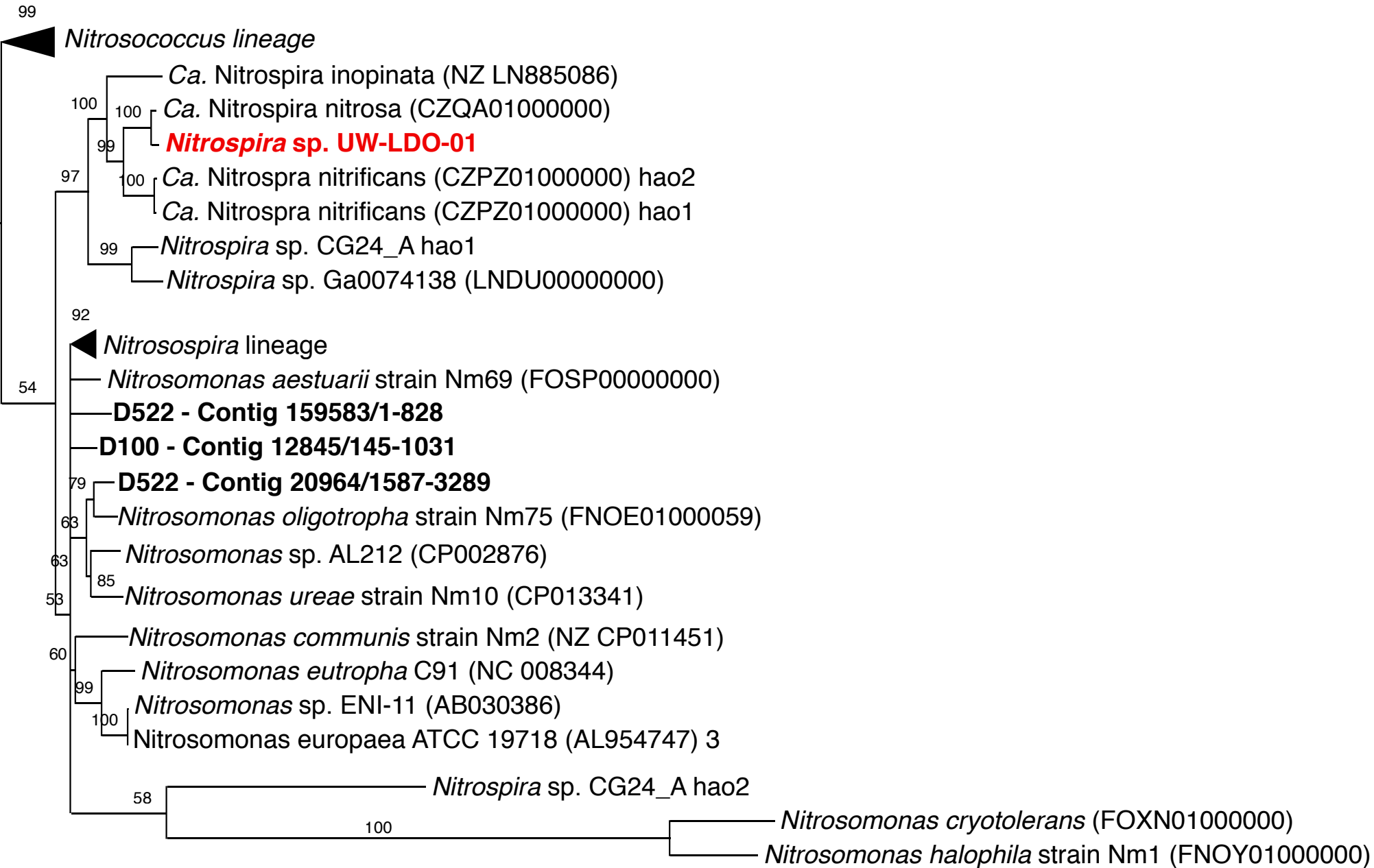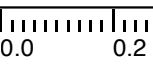

Supplement: FIG S2 [file sys005172133sf5.pdf]

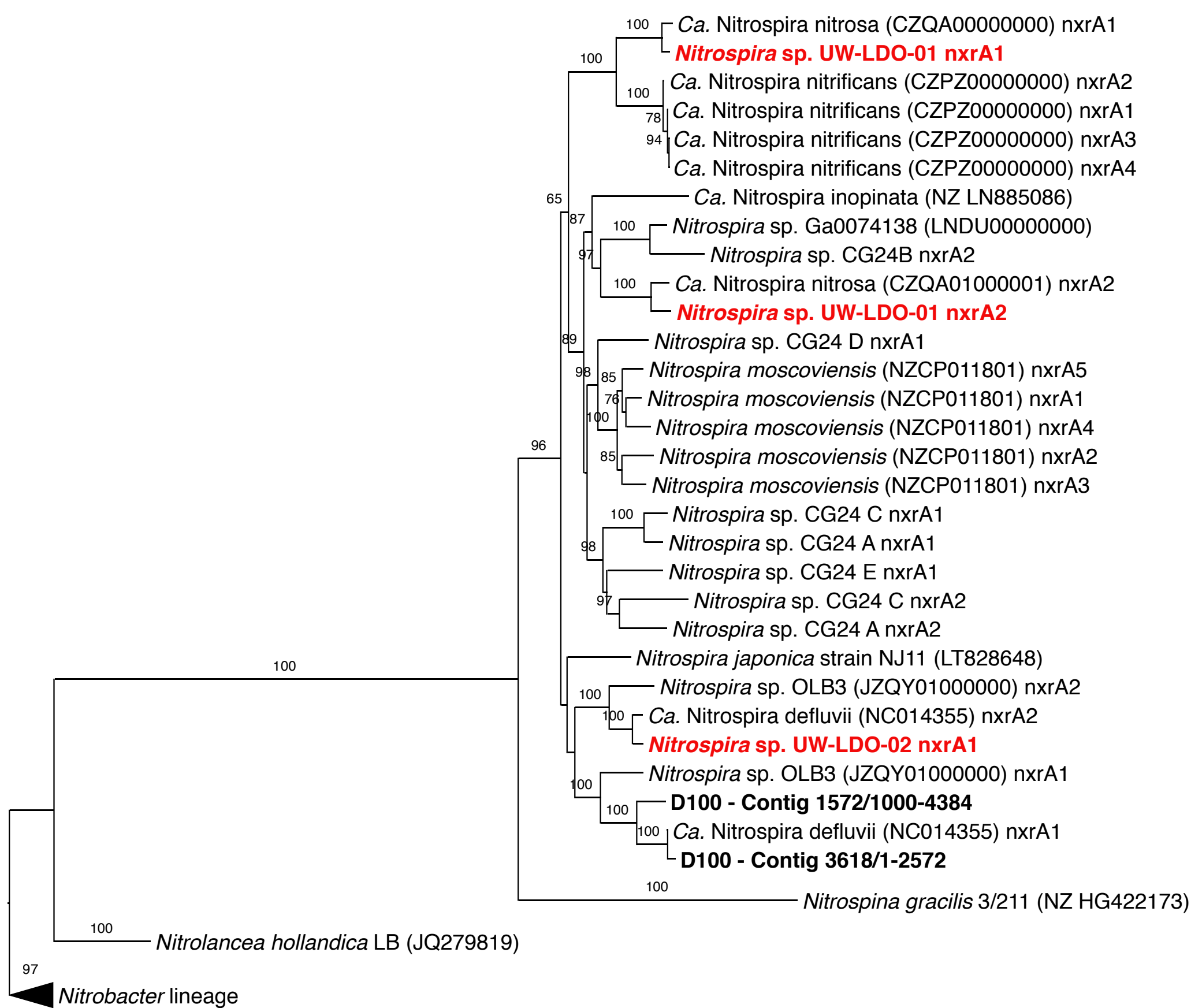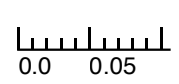

Supplement: FIG S3 [file sys005172133sf6.pdf]

**A**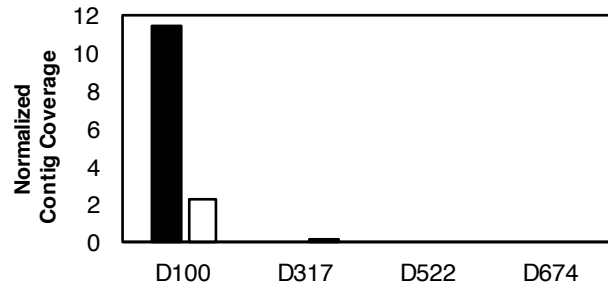**B**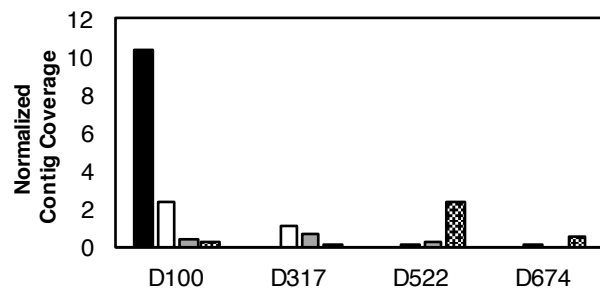**C**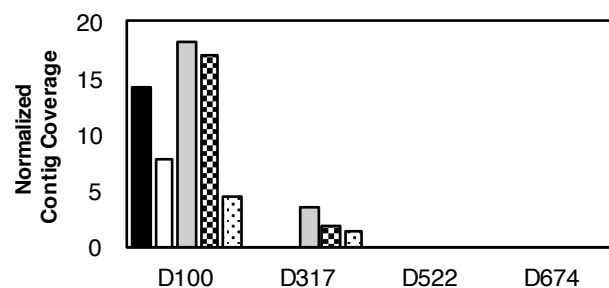

Supplement: FIG S4 [file sys005172133sf7.pdf]
