# Supplementary material for: Genome-Enabled Insights into the Ecophysiology of the Comammox Bacterium “Candidatus Nitrospira nitrosa”
Source: mSystems. 2017 Sep 12;2(5):e00059-17. doi: 10.1128/mSystems.00059-17 (PMC5596200; doi:10.1128/mSystems.00059-17)
Supplement: TABLE S2 [file sys005172133st2.docx]

**Table S2.** Genomes included in the metagenomic mapping analysis and the number of reads mapping to each of them. The number of mapping reads was also normalized by the number of reads in each metagenome, paired-end reads average length and genome size.

|  |  |  | **Metagenomic Sample** | | | | | | | |
| --- | --- | --- | --- | --- | --- | --- | --- | --- | --- | --- |
|  |  |  | **Day** | 100 | **Day** | 317 | **Day** | 522 | **Day** | 674 |
|  |  |  | **Reads Length** | 125 | **Reads Length** | 125 | **Reads Length** | 150 | **Reads Length** | 125 |
|  |  |  | **N^o^ Reads** | 12,630,882 | **N^o^ Reads** | 16,037,438 | **N^o^ Reads** | 109,197,498 | **N^o^ Reads** | 16,056,492 |
| **Functional Group** | **Genome** | **Length (bp)** | **Mapping Reads** | **Normalized Counts** | **Mapping Reads** | **Normalized Counts** | **Mapping Reads** | **Normalized Counts** | **Mapping Reads** | **Normalized Counts** |
| AOA | *Ca.* Nitrosoarchaeum limnia BG20 (AHJG01000000) | 1,855,559 | 4 | 0 | 8 | 0 | 0 | 0 | 0 | 0 |
|  | *Ca.* Nitrosoarchaeum limnia SFB1 (CM001158) | 1,772,718 | 2 | 0 | 2 | 0 | 0 | 0 | 2 | 0 |
|  | *Nitrososphaera viennensis* EN76 (CP007536) | 2,527,938 | 38 | 0 | 48 | 0 | 0 | 0 | 36 | 0 |
|  | *Cenarchaeum symbiosum* A (DP000238) | 2,045,086 | 38 | 0 | 40 | 0 | 0 | 0 | 19 | 0 |
|  | *Nitrosopumilus maritimus* SCM1 (CP000866) | 2,673,040 | 4 | 0 | 2 | 0 | 0 | 0 | 1 | 0 |
|  | *Nitrosopumilus* sp. AR (AJVJ01000001) | 4,084,671 | - | - | 6 | 0 | 0 | 0 | 2 | 0 |
| AOB | *Nitrosococcus oceani* (NC_007484) | 3,481,691 | 992 | 3 | 2,032 | 5 | 21,760 | 9 | 964 | 2 |
|  | *Nitrosococcus mobilis* (FMWO00000000) | 3,082,864 | 8,334 | 27 | 1,834 | 5 | 4,690 | 2 | 259 | 1 |
|  | *Nitrosomonas communis* Nm2 (NZ_CP011451) | 4,067,838 | 686 | 2 | 252 | 0 | 982 | 0 | 66 | 0 |
|  | *Nitrosomonas europaea* (AL954747) | 2,812,094 | 2,702 | 10 | 3,138 | 9 | 10,724 | 5 | 586 | 2 |
|  | *Nitrosomonas eutropha* (NC_008344) | 2,661,057 | 842 | 3 | 316 | 1 | 1,760 | 1 | 96 | 0 |
|  | *Nitrosomonas* sp. AL212 (NC_015222) | 3,180,526 | 910 | 3 | 340 | 1 | 6,868 | 3 | 287 | 1 |
|  | *Nitrosomonas* sp. Is79A3 (CP002876) | 3,783,444 | 2,678 | 7 | 1,346 | 3 | 10,166 | 4 | 330 | 1 |
|  | *Nitrosomonas ureae* Nm10 (CP013341) | 3,307,820 | 810 | 2 | 214 | 1 | 2,512 | 1 | 99 | 0 |
|  | *Nitrosomonas aestuarii* (FOSP01000000) | 3,994,034 | 594 | 1 | 434 | 1 | 1,144 | 0 | 90 | 0 |
|  | *Nitrosomonas halophile* (FNOY01000000) | 1,645,259 | 880 | 5 | 610 | 3 | 1,366 | 1 | 167 | 1 |
|  | *Nitrosomonas nitrosa* (FOUF01000000) | 3,154,865 | 400 | 1 | 268 | 1 | 808 | 0 | 51 | 0 |
|  | *Nitrosospira briensis* (NZ_CP012371) | 3,441,569 | 630 | 2 | 432 | 1 | 984 | 0 | 146 | 0 |
|  | *Nitrosospira multiformis* (CP000103) | 3,184,243 | 614 | 2 | 408 | 1 | 844 | 0 | 111 | 0 |
|  | *Nitrosospira* sp. NpAV (JXQM01000000) | 3,210,113 | 1,096 | 3 | 464 | 1 | 1,108 | 0 | 150 | 0 |
| Comammox | *Nitrospira* sp. UW-LDO-01 | 3,909,139 | 545,070 | 1,380 | 244 | 0 | 990 | 0 | 105 | 0 |
|  | *Nitrospira* sp. Ga0074138 (LNDU01000000) | 4,111,525 | 2,980 | 7 | 302 | 1 | 26 | 0 | 71 | 0 |
|  | *Ca.* Nitrospira nitrosa (CZQA01000000) | 4,422,398 | 41,490 | 93 | 188 | 0 | 88 | 0 | 48 | 0 |
|  | *Ca.* Nitrospira nitrificans (CZPZ01000000) | 2,385,067 | 4,766 | 20 | 410 | 1 | 86 | 0 | 108 | 0 |
|  | *Ca.* Nitrospira inopinata (LN885086) | 3,155,724 | 1,628 | 5 | 360 | 1 | 278 | 0 | 125 | 0 |
| NOB | *Nitrospira moscoviensis* NSP_M-1 (NZ_CP011801) | 4,589,485 | 1,852 | 4 | 644 | 1 | 82 | 0 | 253 | 0 |
|  | *Nitrospira_defluvii* (NC_014355) | 4,317,083 | 395,170 | 906 | 113,576 | 205 | 8,268 | 3 | 38,506 | 69 |
|  | *Nitrospira* sp. UW-LDO-02 | 3,541,761 | 771,620 | 2,157 | 101,916 | 224 | 4,562 | 2 | 18,577 | 41 |
|  | *Nitrobacter winogradskyi* (NC_007406) | 3,402,093 | 902 | 3 | 1,066 | 2 | 9,950 | 4 | 759 | 2 |
|  | *Nitrobacter hamburgensis* (NC_007964) | 4,406,967 | 1,784 | 4 | 1,744 | 3 | 16,432 | 5 | 1,018 | 2 |
|  | Nitrospira sp. OLB23 (JZQY00000000) | 3,754,263 | 6,276 | 17 | 2,086 | 4 | 2,218 | 1 | 516 | 1 |
| Anammox | Ca. Brocadia fulgida (LAQJ01000000) | 3,552,939 | 36 | 0 | 40 | 0 | 78 | 0 | 21 | 0 |
|  | Ca. Brocadia caroliniensis (AYTS01000000) | 3,728,197 | 20 | 0 | 42 | 0 | 6 | 0 | 16 | 0 |
|  | Ca. Kuenenia stuttgartiensis (AMCF01000000) | 3,810,257 | 154 | 0 | 148 | 0 | 46 | 0 | 339 | 1 |
|  | Ca. Brocadia sinica (BAFN01000000) | 4,077,002 | 28 | 0 | 32 | 0 | 46 | 0 | 15 | 0 |
|  | Ca. Jettenia caeni (BAFH01000000) | 4,087,350 | 20 | 0 | 32 | 0 | 22 | 0 | 12 | 0 |
|  |  | **Total** | **1,796,050** | **4,667** | **234,730** | **476** | **108,696** | **43** | **63,548** | **126** |
