# Supplementary material for: Genome-Enabled Insights into the Ecophysiology of the Comammox Bacterium “Candidatus Nitrospira nitrosa”
Source: mSystems. 2017 Sep 12;2(5):e00059-17. doi: 10.1128/mSystems.00059-17 (PMC5596200; doi:10.1128/mSystems.00059-17)
Supplement: TABLE S3 [file sys005172133st3.docx]

**Table S3.** Inventory of genes involved in energy-driving processes from complete and draft genomes of *Nitrospira*. Grey and white rectangles represent presence and absence of each gene, respectively. Numbers represent the number of copies of multiple-copy genes encoded in each genome.

|  |  |  | Comammox | NOB |
| --- | --- | --- | --- | --- |

| **Category** | **Gene** | **Function** | ***Nitrospira* sp. UW-LDO-01** | ***Ca.* Nitrospira nitrosa** | ***Ca.* Nitrospira nitrificans** | ***Ca.* Nitrospira inopinata** | ***Nitrospira* sp. Ga0074138** | ***Nitrospira* sp. UW-LDO-02** | ***Nitrospira moscoviensis*** | ***Nitrospira defluvii*** | ***Nitrospira* sp. OL23** |
| --- | --- | --- | --- | --- | --- | --- | --- | --- | --- | --- | --- |

| Ammonia Oxidation | *amoA* | Ammonia monooxygenase subunit alpha |  | 2 |  |  |  |  |  |  |  |
| --- | --- | --- | --- | --- | --- | --- | --- | --- | --- | --- | --- |
|  | *amoB* | Ammonia monooxygenase subunit beta |  |  |  |  |  |  |  |  |  |
|  | *amoC* | Ammonia monooxygenase subunit gamma | 2 | 3 | 4 | 3 | 3 |  |  |  |  |
| Hydroxylamine Oxidation | *haoA* | Hydroxylamine oxidoreductase subunit alpha |  |  | 21 |  |  |  |  |  |  |
|  | *haoB* | Hydroxylamine oxidoreductase subunit beta |  |  |  |  |  |  |  |  |  |
|  | *cycA* | Cytochrome c protein c554 |  |  |  |  |  |  |  |  |  |
|  | *cycB* | Cytochrome c protein, cM552 |  |  |  |  |  |  |  |  |  |
| Nitrite Oxidation | *nxrA* | Nitrite oxidoreductase, alpha subunit |  | 2 | 4 |  |  |  | 5 | 2 | 2 |
|  | *nxrB* | Nitrite oxidoreductase, beta subunit |  | 2 | 3 |  |  |  | 4 | 2 | 2 |
| Nitrite Reduction | *nirK* | Copper- containing dissimilatory nitrite reductases |  |  |  |  |  |  | 2 |  |  |
|  | *nrfA* | cytochrome c nitrite reductase |  |  |  |  |  |  |  |  |  |
| Urea Hydrolysis | *urtA* | Urea ABC transporter | 1 | 1 | 1 | 1 | 1 |  | 1 |  |  |
|  | *urtB* | Urea ABC transporter | 1 | 1 | 1 | 1 | 1 |  |  |  |  |
|  | *urtC* | Urea ABC transporter | 1 | 1 | 1 | 1 | 1 |  |  |  |  |
|  | *urtD* | Urea ABC transporter | 1 | 1 | 1 | 1 | 1 |  |  |  |  |
|  | *urtE* | Urea ABC transporter | 1 | 1 | 1 | 1 | 1 |  |  |  |  |
|  | *ureA* | Urease subunit gamma | 1 | 1 | 1 | 1 | 1 |  | 1 |  |  |
|  | *ureB* | Urease subunit beta | 1 | 1 | 1 | 1 | 1 |  | 1 |  |  |
|  | *ureC* | Urease subunit alpha | 1 | 1 | 1 | 1 | 1 |  | 1 |  |  |
|  | *ureD* | Urease accessory protein | 1 | 1 | 1 | 1 | 1 |  | 1 |  |  |
|  | *ureF* | Urease accessory protein | 1 | 1 | 1 | 1 | 1 |  | 1 |  |  |
|  | *ureG* | Urease accessory protein | 1 | 1 | 1 | 1 | 1 |  | 1 |  |  |
| Cyanase degradation | *cynS* | Cyanase hydratase |  |  |  |  |  | 1 | 1 | 1 | 1 |
| Formate Oxidation | *fdsA* | Formate dehydrogenase subunit alpha |  |  |  |  |  | 1 | 1 | 1 | 1 |
|  | *fdsB* | Formate dehydrogenase subunit beta |  |  |  |  |  | 1 | 1 | 1 | 1 |
|  | *fdsC* | Formate dehydrogenase subunit gamma |  |  |  |  |  | 1 | 1 | 1 | 1 |
|  | *focA* | Formate transporter |  |  |  |  |  | 1 | 1 | 1 | 1 |
| Hydrogen Oxidation | *hoxA* | Hydrogenase transcriptional regulatory protein |  |  |  |  |  |  | 1 |  |  |
|  | *hupS* | Putative [NiFe] hydrogenase small subunit |  |  |  |  |  |  | 1 |  |  |
|  | *hupL* | Putative [NiFe] hydrogenase large subunit |  |  |  |  |  |  | 1 |  |  |
|  | *hypA* | Hydrogenase nickel incorporation protein | 1 | 1 | 1 | 1 | 1 |  | 1 |  |  |
|  | *hypB* | Hydrogenase nickel incorporation protein | 1 | 1 | 1 | 1 | 1 |  | 1 |  |  |
|  | *hypC* | Hydrogenase expression/formation protein | 1 | 1 | 1 | 1 | 1 |  | 1 |  |  |
|  | *hypD* | Hydrogenase expression/formation protein | 1 | 1 | 1 | 1 | 1 |  | 1 |  |  |
|  | *hypE* | Hydrogenase maturation protein | 1 | 1 | 1 | 1 | 1 |  | 1 |  |  |
|  | *hypF* | Hydrogenase metallocenter assembly protein |  |  |  |  |  |  | 1 |  |  |
|  | *hyfB* | Hydrogenase, membrane subunit HyfB | 1 | 1 |  |  | 1 | 1 | 1 | 1 | 1 |
|  | *hyfC* | Hydrogenase, membrane subunit HyfC | 1 | 1 |  |  | 1 | 1 | 1 | 1 | 1 |
|  | *hyfE* | Hydrogenase, membrane subunit HyfE | 1 | 1 |  |  | 1 | 1 | 1 | 1 | 1 |
|  | *hyfF* | Hydrogenase, membrane subunit HyfF | 1 | 1 |  |  | 1 | 1 | 1 | 1 | 1 |
|  | *hyfG* | Hydrogenase, large subunit HyfG | 1 | 1 |  |  | 1 | 1 | 1 | 1 | 1 |
|  | *hyfI* | Hydrogenase, small subunit HyfI | 1 | 1 |  |  | 1 |  | 1 | 1 | 1 |
| Sulfur reduction | *hydA* | Sulfhydrogenase, subunit alpha | 1 | 1 | 1 | 1 | 1 |  |  |  |  |
|  | *hydB* | Sulfhydrogenase, subunit beta | 1 | 1 | 1 | 1 | 1 |  |  |  |  |
|  | *hydD* | Sulfhydrogenase, subunit delta | 1 | 1 | 1 | 1 | 1 |  |  |  |  |
|  | *hydG* | Sulfhydrogenase, subunit gamma | 1 | 1 | 1 | 1 | 1 |  |  |  |  |
|  | *hybD* | Hydrogenase maturation preotease | 1 | 1 | 1 | 1 | 1 |  |  |  |  |
